# Editorial: Bacterial wilt: pathogenic mechanism, disease control, bacteria-plant and bacteria-environmental microorganism interactions

**DOI:** 10.3389/fmicb.2023.1322189

**Published:** 2023-11-22

**Authors:** Peng Li, Yong Zhang, Yufan Chen

**Affiliations:** ^1^Ministry of Education Key Laboratory for Ecology of Tropical Islands, Hainan Provincial Key Laboratory for Tropical Plant and Animal Ecology, College of Life Sciences, Hainan Normal University, Haikou, China; ^2^College of Resources and Environment, Southwest University, Chongqing, China; ^3^Key Laboratory of Chinese Medicinal Resource From Lingnan, Research Center of Chinese Herbal Resource Science and Engineering, Ministry of Education, Joint Laboratory of National Engineering Research Center for the Pharmaceutics of Traditional Chinese Medicines, Guangzhou University of Chinese Medicine, Guangzhou, China

**Keywords:** bacterial wilt, disease control, bacteria-plant interaction, type III secretion system, effectors

In the Research Topic *Bacterial wilt: pathogenic mechanism, disease control, bacteria–plant and bacteria–environmental microorganism interactions*, we aimed to collect manuscripts not just limited to the studies of *Ralstonia solanacearum* but also welcomed all the new progress of *Enterobacter, Erwinia, Kosakonia*, and *Xanthomonas*. Notably, the five accepted papers have focused on the wilt disease caused by *R. solanacearum*.

Type III secretion system (T3SS) is one of the most important virulence systems in *R. solanacearum*. The transcription of T3SS is controlled by HrpB, HrpG, PrhG, and PhcA. Zhang et al. demonstrated that two 3-dehydroquinases, AroQ1 and AroQ2, are cooperatively essential for aromatic amino acids biosynthesis via the shikimate pathway, and they also promote T3SS expression by taking the well-characterized PrhA signaling cascade. Altogether, the contribution of AroQ1/2 to pathogenicity was partially due to insufficient SA inside host plants, and the involvement of AroQ1/2 on T3SS expression was mediated through the PrhA signaling cascade to HrpG and HrpB.

The *R. solanacearum* can use T3SS to inject type III effectors (T3Es) into host cytosol that subvert the host defense to facilitate bacterial survival. Miao et al. conducted yeast two-hybrid and pull-down assays and then identified one T3E (RipAA) that could induce the accumulation of hydrogen peroxide and genome DNA degradation in *Nicotiana benthamiana*. They demonstrated that the marker genes for salicylic acid signaling were induced and that those for jasmonic acid signaling were reduced by RipAA. Moreover, the RipAA could interact with chloroplastic AtpB, and the silencing of *atpB* resulted in the inability to induce hypersensitive response and an enhanced sensitivity to bacterial wilt.

Results have indicated that T3Es contribute to both virulence on susceptible hosts and cause immune response in non-host plants by coupling the cognate receptor. The article by Ouyang et al. addressed the role of the E3 ligase activity-dependent manner in RipAW-triggered plant immunity. When the RipAW was mutated, it could not induce cell death but retained the ability of triggering plant immunity in *N. benthamiana*. In addition, the E3 ligase activity was not essential for RipAW-triggered plant immunity, while the RipAW- and RipAWC177A-triggered immunity in *N. benthamiana* required SGT1, shedding new light on effector-triggered plant immunity.

Developing environmentally friendly and economically viable strategies for controlling bacterial wilt is extremely necessary. Xia et al. screened 100 plant-derived compounds that are antagonistic against *R. solanacearum*; of these, 12 compounds, including harmine, harmine hydrochloride, citral, vanillin, and vincamine, could suppress *R. solanacearum* growth in a liquid medium with an inhibition rate > 50%. They mainly focused on the inhibitory characteristics of harmine; when treated with its minimum inhibitory concentration (120 mg/L) for 2 h, it could kill more than 90% of *R. solanacearum*, which could, in turn, suppress the expression of virulence-related gene *xpsR*, inhibit biofilm formation, and reduce disease development in tobacco and tomato plants.

Biological control offers numerous advantages. Sun et al. demonstrated that treatment with *Bacillus subtilis* R31 significantly reduced the incidence of tomato bacterial wilt as R31 could directly inhibit the growth of R. solanacearum by taking lipopeptides. They also found that R31 could stably colonize the rhizosphere soil and root tissues of tomato plants, reduce the *R. solanacearum* population in the rhizosphere soil, and alter the microbial community that interacts with *R. solanacearum*.

This Research Topic will pave the way for dealing with this devastating disease and developing effective control methods for bacterial wilt prevention and control.

## Author contributions

PL: Writing – original draft. YZ: Writing – review & editing. YC: Writing – review & editing.

